# Environmental Regulation, Technological Innovation, and Export Competitiveness: An Empirical Study Based on China’s Manufacturing Industry

**DOI:** 10.3390/ijerph17041427

**Published:** 2020-02-23

**Authors:** Jiayue Liu, Jing Xie

**Affiliations:** 1 School of Economics, South-Central University for Nationalities, Wuhan 430074, China; h0505@foxmail.com; 2 Hubei Moderately Prosperous Society in all respects Construction Research Institute, Wuhan 430074, China; 3 School of Economics, Zhongnan University of Economics and Law, Wuhan 430073, China

**Keywords:** environmental regulation, technological innovation, export value added, revealed comparative advantage, export competitiveness, Chinese manufacturing

## Abstract

A current and universal challenge, particularly in developing nations, is the establishment of effective environmental regulation policies that protect the ecological environment without adversely affecting the international competitiveness of the domestic manufacturing industry. To deal with this dilemma, this study investigates the export competitiveness of China’s manufacturing industry from the viewpoint of export value added. The Porter hypothesis is applied for an empirical investigation of the effect of environmental regulation on export competitiveness and to determine the presence of intra-industry heterogeneity. Furthermore, this study seeks to understand the mechanisms through which environmental regulation affects export competitiveness by exploring the two main approaches to technological innovation. The findings reveal that environmental regulation has a promotion effect of approximately 2% on the export competitiveness of China’s manufacturing industry; however, this effect is non-linear and displays a “U-shaped” tendency, indicating that certain prerequisites must be fulfilled to validate the Porter hypothesis. In addition, the effect of environmental regulation displays significant intra-industry heterogeneity, which is evident primarily in heavily polluting sub-industries and to a lesser extent in moderately polluting sub-industries but insignificant in lightly polluting sub-industries. Environmental regulation also differs significantly in the mechanisms through which it affects different approaches to technological innovation. Independent research and development is affected by environmental regulation through the compliance cost effect, which limits export competitiveness, while technology introduction is affected by the innovation offset effect, which favors export competitiveness. These findings offer political implications for the sustainable development of the ecological environment and foreign trade.

## 1. Introduction

Amid economic integration and market globalization, Chinese products have rapidly penetrated into overseas markets due to their inherent advantages. In 2015, China’s commodity exports contributed to nearly 14% of the international market and totaled US$ 2270 billion, 94% of which accounted for manufactured goods. “Made in China” has become one of the most recognized trademarks in the international market. Nevertheless, several concerns have arisen from China’s emergence as a manufacturing power. First, poor air quality in many Chinese regions illustrates the immense environmental cost of China’s economic growth. Second, under a trade-development model centered around low-quality and low-price products, the “Made in China” model results in low-end lock-in in the value chain of the global manufacturing industry [1], resulting in the continuous deterioration of China’s terms of trade [2]. As China’s economic development stabilizes, constraints on resources and the ecological environment have increased, contributing to an escalation of factor costs. These changes in the socio-economic landscape have presented challenges to the transformation and development of China’s manufacturing industry. The Chinese government is increasingly focused on environmental protection and rigorous environmental governance. The 13th Five-Year Plan for Ecological Environmental Protection established the target of improving the overall quality of the ecological environment through implementing an environmental protection system with stringent standards. Moreover, the Chinese government promulgated the Made in China 2025 plan in 2015 and called for innovative and green development to create new competitive advantages in exports and to accomplish the strategic goal of transforming the Chinese manufacturing industry into a world leader. However, there is considerable debate as to whether stringent environmental governance positively influences trade competitiveness or whether it limits the comparative advantage of Chinese exports. As China is currently at a crucial stage of economic transformation, an analysis of the effect of environmental regulation on trade competitiveness has important implications for both the sustainable development of the ecological environment and foreign trade.

Environmental regulation is a series of pollution control policies, such as laws and regulations that are established to support the sustainable development of the ecological environment. They constrain the pollution emissions of enterprises from the production processes and motivate firms to engage in green production. Existing research shows that environmental regulation generates additional production costs for enterprises, which potentially limit technological innovation, thus adversely affecting the international competitiveness of enterprises [3,4]; however, environmental regulation also induces the innovation offset effect, whereby the benefits of innovation can offset the cost of environmental regulation, motivating enterprises to strengthen technological innovation to enhance their competitiveness [5,6]. Thus, two contrasting conclusions exist regarding the effect of environmental regulation on export competitiveness. A possible explanation is that technological innovation can be divided into two approaches: independent research and development (R and D) and technology introduction. Independent R and D refers specifically to internal R and D expenditure and the expenditure of digestion and absorption, and technology introduction mainly includes the expenditure on the introduction or acquisition of foreign and domestic technology [7]. In general, enterprises can obtain faster returns on innovation performance through technology introduction than independent R and D. Therefore, the effects of environmental regulation differ for the two approaches. Additionally, it is possible that the effect of environmental regulation on competitiveness depends on an enterprise’s approach to technological innovation. Existing literature largely examines export competitiveness using traditional comparative advantage indices such as revealed comparative advantage (RCA). With the gradual deepening of global vertical specialization, international specialization is distributed across various production processes of the same product. Exports, therefore, contain a certain import content, and the resulting gains from trade only reflect the value added of the production process in which it is involved. As traditional comparative advantage indices are based on the overall value of export products, they do not provide a realistic measure of export competitiveness in this context.

This study first investigates the export competitiveness of China’s manufacturing industry from the viewpoint of export value added. The Porter hypothesis is revisited for an empirical investigation of the effects of environmental regulation on export competitiveness and to identify intra-industry heterogeneity, using panel data of 27 sub-industries in China’s manufacturing industry from 2000 to 2014. The mechanisms through which environmental regulation affects export competitiveness are also examined based on the independent R and D and technology introduction approaches to technological innovation. The findings reveal that environmental regulation has a promotion effect of approximately 2% on the export competitiveness of China’s manufacturing industry. However, this effect is non-linear and displays a “U-shaped” relationship, indicating that certain prerequisites must be fulfilled to validate the Porter hypothesis. In addition, the promotion effect of environmental regulation differs significantly within industries, providing a substantial effect on heavily and moderately polluting sub-industries but an insignificant effect on lightly polluting sub-industries. Environmental regulation also differs significantly in the mechanisms through which it affects the two different approaches to technological innovation. Independent R and D is primarily influenced by the cost effect, which tends to limit its export competitiveness, while technology introduction is influenced by the innovation offset effect, which tends to favors export competitiveness.

This study provides two important contributions to the existing literature. First, in contrast to the methods used in previous research, this study applies comparative advantage indices measured by export value added and, therefore, may reflect a more realistic measure of the international competitiveness of export products in the context of vertical specialization and trade. Second, this study expands on the Porter hypothesis and provides an in-depth analysis of the mechanisms through which environmental regulation affects export competitiveness through the two main approaches to technological innovation.

The remainder of this paper is organized as follows. Section 2 presents the literature review. Section 3 describes the materials and methods used in this study. The empirical results are presented in Section 4. Section 5 discusses the empirical results in detail. The main conclusions and limitations of this study are shown in the last section.

## 2. Literature Review

### 2.1. Environmental Regulation and Export Performance

Two contrasting views are provided within the existing literature on environmental regulation and export competitiveness. The traditional perspective argues that environmental regulation curtails the international competitiveness of a country’s export products by increasing production costs and inhibiting technological innovation. To ensure that production activities adhere to government policies on environmental regulation, enterprises must either control their pollution emissions or boost their technical standards of pollution governance, which inevitably increases their production costs. This increase in expenditure can lead to the reallocation of resources and a reduction in investment in technological innovation, thus imposing constraints on competitiveness [8]. Mani and Wheeler [3] and Ederington et al. [4] discovered that high-standard environmental regulation diminishes the comparative advantage of the respective industry of a country and exerts a certain influence on trade patterns. Feiock and Rowland [9] and Cagatay and Mihci [10] conducted an empirical analysis and revealed a significant negative correlation between the environmental regulation stringency of a country and its export trade. In contrast, other scholars support the Porter hypothesis, which holds that an increase in the stringency of a country’s environmental regulation does not lower its international competitiveness; moreover, it compels enterprises to undertake technological innovation. In the long term, the offset effect of such regulation favors international competitiveness [5]. Jaffe and Palmer [11] carried out an empirical study on the U.S. manufacturing industry and found that environmental regulation has a significant positive effect on R and D expenditure. Ramanathan et al. [12] harnessed data of U.K. and Chinese enterprises to examine the relationship between environmental regulation and technological innovation, and the results aligned with the Porter hypothesis. Costantini and Mazzanti [6] found that the innovation offset effect prevented stringent environmental regulations from weakening the international competitiveness of the manufacturing industry across European Union countries. Wang et al. [13] also drew a similar conclusion using industrial data of Organization for Economic Cooperation and Development (OECD) countries; however, they also found that the stringency of environmental regulation is subject to a certain threshold, beyond which the cost effect exceeds the innovation offset effect.

Furthermore, recently some researchers have paid close attention to the techno-environmental impact of the circular economy, which is an appealing administrative tool among governments and enterprises [14]. Kyriakopoulos et al. [15] analyzed the action of technologies on sewage disposal, organic waste treatment, agrarian development, and food waste in the context of circular economy. Aravossis et al. [16] argued that it is necessary to develop a holistic analysis on the evaluation of the best production practices and the performance of industrial organization, based on the principles of the circular economy. Therefore, with the implementation of government policies on environmental regulation, the clean-technology input may be increased in manufacturing industry, and then the performance of holistic assessment in industry is improved. Additionally, there are other studies that focus on the export performance of agriculture and service industry. Morkūnas et al. [17] and Smutka et al. [18] examined the effects of policies on the trade performance of agricultural industry goods. Gulicheva et al. [19] analyzed the relationship between innovative education environment and the export competitiveness of Russian education services.

With the introduction of increasingly stringent policies to address China’s environmental issues, scholars have begun to ponder the effect of environmental regulation on technological innovation and trade competitiveness. Li and Zhu [20], Bu et al. [21], and Shen et al. [22] found in their empirical studies that China’s environmental regulation policies validate the Porter hypothesis. Li et al. [23] employed panel data of China’s industrial sector from 1998 to 2008 to conduct an empirical analysis based on three comparative advantage indices. The findings suggest that environmental regulation promotes comparative advantage in the industrial sector but manifests a threshold effect, indicating an inverted “U-shaped” relationship between environmental regulation and comparative trade advantage. In a study based on data of Chinese microenterprises, Li and Chen [24] revealed that the enforcement of environmental regulation policies stifles China’s green total factor productivity in the short term; however, long-term environmental regulation results in a win-win situation between environmental protection and enterprises’ competitiveness. However, some studies draw the opposite conclusion. Tu et al. [25] conducted an empirical survey on 232 Chinese cities using difference-in-differences estimation and discovered that China’s pollution levy standards reform inhibits growth in green total factor productivity. Ren and Huang [26] made use of bilateral trade data between China and 37 trading partners and examined the effect of environmental regulation of China’s export trade, using the extended gravity model. The results indicate a significant negative correlation between the stringency of China’s environmental regulation and its export trade, identifying environmental regulation as a major determinant of comparative trade advantage. Wu et al. [27] found that environmental regulation does not enhance the innovation capacity of China’s manufacturing industry.

In sum, the literature demonstrates that environmental regulation can significantly affect the export competitiveness of a country or an industry through the cost effect and the offset effect on technological innovation and that the relationship is likely to be non-linear.

### 2.2. Measure of Export Competitiveness

RCA, as proposed by Balassa [28], is an index widely applied to research on the theory of comparative advantage and international competitiveness. The RCA index reflects a country’s comparative advantage for a certain product based on the share of this product in the gross exports of the country and the world. It is applicable to conventional inter- and intra-industrial terms of trade, with the basic rationale that if a product occupies a larger share of gross national exports, it is expected to have a relatively higher level of labor productivity. If a country’s share of a product exceeds the global export share of other countries for that product, the country will hold a greater comparative advantage and benefit from stronger international competitiveness. In addition, the Global Competitiveness Index provided by the World Economic Forum is used as a proxy for the global competitiveness at national level [29]. Simionescu [30] estimated the competitiveness of Romania at the regional level based on dynamic panel data. Nwabueze and Mileski [31] analyzed the competitive advantage from a new perspective of effective communication at the enterprise level.

Scholars have recently started re-examining comparative advantage in the context of vertical specialization and terms of trade [32], using export value added indices as a new direction for research. Timmer et al. [33] identified RCA as the standard instrument for analyzing specialization and competitive advantages; in the case of vertical specialization, RCA analysis retains its usefulness, though with a different interpretation. Beaudreau [34] also posited that data derived from export value added offers a more accurate measure of a country’s advantages and disadvantages. In China, domestic studies on industrial international competitiveness have also begun to focus on export value added [35,36,37]. The studies differ in focal points and yield important outcomes; however, few studies have analyzed RCA using export value added to examine the evolution of the export competitiveness of China’s manufacturing industry. Moreover, differences in the statistical scope and measurement methods for domestic value added have led to variations in the results of existing measurements.

The literature cited above offers several perspectives and provides a valuable source of information for elucidating the relationship between environmental regulation and export competitiveness. It is, however, important to note that most existing studies provide further validation or extension of the Porter hypothesis and do not consider the varying approaches to technological innovation. Within the constraints of environmental regulation, enterprises have differential preferences for the method applied to technological innovation, and few studies address the contradictions in existing conclusions from this perspective. In addition, existing studies on export competitiveness largely focus on traditional comparative advantage indices, which are not appropriate to study vertical specialization and terms of trade and are unable to realistically reflect a country’s export competitiveness. For China and other countries with a manufacturing industry that contributes substantially to gross exports, international competitive advantage and gains from trade should be delineated realistically based on export value added rather than gross exports. Therefore, this study examines the effect of environmental regulation on the export competitiveness of China’s manufacturing industry from the standpoint of export value added and highlights the mechanisms in which environmental regulation affects technological innovation strategies. The findings are expected to assist in China’s selection of suitable environmental regulation policies, thus promoting the sustainable development of the ecological environment and economic trade.

## 3. Materials and Methods

### 3.1. Econometric Model

Existing studies suggest that environmental regulation may exhibit a non-linear effect on export competitiveness; therefore, the quadratic term of environmental regulation is introduced to the model for this study. The econometric model is as follows:

(1) $$\ln VRCA_{jt}=\alpha_{0}+\alpha_{1}\ln ER_{jt}+\alpha_{2}{\left(\ln ER_{jt}\right)}^{2}+\psi X+\delta_{j}+\varepsilon_{jt}$$

where ln denotes the natural logarithm applied to a variable to produce more stationary data; *j* represents the manufacturing industry; *t* stands for the year; *VRCA* is RCA measured by export value added to reflect the export competitiveness of the manufacturing industry; *ER* represents environmental regulation; δ denotes industry fixed effect, which reflects the intra-industrial differences in characteristics; ε is the random error term; and *X* denotes the five control variables that may influence export competitiveness: technological innovation, capital intensity (*Capital*), human capital (*Human*), size of the enterprise (*Size*) and foreign direct investment (*FDI*). Enterprises may choose between two different approaches to technological innovation [7]; therefore, *Innovation* is divided into independent R and D (*RD*) and technology introduction (*TI*) to investigate their respective effects on export competitiveness.

Furthermore, to examine the different mechanisms through which environmental regulation acts on export competitiveness, interaction terms between environmental regulation and the two technological innovation approaches are introduced into Equation (1), resulting in the following econometric model:

(2) $$\begin{array}{cc} \ln VRCA_{jt} & =\beta_{0}+\beta_{1}\ln ER_{jt}+\beta_{2}\ln ER_{jt}\times \ln RD_{jt}+\beta_{3}\ln ER_{jt}\times \ln TI_{jt}\\ & +\beta_{4}{\left(\ln ER_{jt}\right)}^{2}+\psi X+\delta_{j}+\mu_{jt} \end{array}$$

where $\ln ER_{jt}\times \ln RD_{jt}$ represents the interaction term of environmental regulation and independent R and D. If $\beta_{2}$ is positive, environmental regulation produces an innovation offset effect on independent R and D and favors export competitiveness, while if $\beta_{2}$ is negative, environmental regulation mainly exerts a cost effect on independent R and D and inhibits export competitiveness. $\ln ER_{jt}\times \ln TI_{jt}$ denotes the interaction term of environmental regulation and technology introduction, and $\beta_{3}$ carries similar economic implications as $\beta_{2}$ and can be interpreted in the same way.

### 3.2. RCA Based on Export Value Added (VRCA)

Recently, scholars have enriched relevant research on export value added and have developed increasingly precise measurement methods. Major academic and statistical institutes worldwide, such as the OECD, the World Trade Organization, the United Nations Statistics Division, Eurostat, the Japan Economic Research Institute, and Purdue University in the U.S, have progressively established relevant statistical systems and databases. The measurement of export value added can be summarized by three aspects: the domestic value added of an industry in export value of products, the domestic value added in reimported intermediate goods used in the production of export products, and factor income from abroad in domestic value added. The third aspect is concerned with whether the input-output and value added of a country’s production are based on the place of production or ownership [33]. As estimations of export value added using an input-output table usually give no consideration to the ownership of export products, the calculations in this study are premised on the location of production; they involve the direct and indirect domestic value added of an industry in the products manufactured in and exported from China, as well as the domestic value added embodied in reimported intermediate goods. Export products are not divided into intermediate and final products.

By drawing the methodology adopted by Koopman et al. [38], this study calculates the export value added using the input-output model. The derived equation is as follows:

(3) $$VE_{j}=v_{j}{\left(I-A^{d}\right)}^{-1}E_{j}+RIM_{j}$$

where *j* represents the industry, *VE* denotes a vector of export value added, *v* is a vector of the direct value added of an industry, ${\left(I-A^{d}\right)}^{-1}$ represents the Leontief inverse matrix of the domestic intermediate inputs of an industry, *E* denotes the export vector, and *RIM* is a vector of the domestic value added content of re-imported intermediate goods in exports.

Balassa [28] proposed the following equation for measuring the RCA index:

(4) $$RCA_{j}^{i}=\frac{E_{j}^{i}/E^{i}}{E_{j}^{w}/E^{w}}$$

where *i* denotes the country or region, $E_{j}^{i}$ represents the export value of industry *j* in country *i*, $E^{i}$ is the gross exports of country *i*,$E_{j}^{w}$ denotes the gross world exports of industry *j*, and $E^{w}$ represents gross world exports. 

With reference to Equation (4), the export value is substituted by the export value added to obtain the RCA based on export value added (*VRCA*). The equation is as follows:

(5) $$VRCA_{j}^{i}=\frac{VE_{j}^{i}/VE^{i}}{VE_{j}^{w}/VE^{w}}$$

where $VRCA_{j}^{i}$ represents the RCA based on export value added of country *i*’s industry *j*,$VE_{j}^{i}$ denotes the export value added of country *i*’s industry *j*,$VE^{i}$ is the total export value added of country *i*, $VE_{j}^{w}$ is the world’s total export value added of industry *j*, and $VE^{w}$ is the world’s total export value added. Using the *VRCA* index, changes in the international competitiveness of a country’s industry can be tracked from the viewpoint of export value added. Compared with the traditional comparative advantage indices, such as trade competitiveness (TC) index and RCA index, which may distort the actual export competitiveness, the *VRCA* index can factually indicate how much a country or industry gains from foreign trade. Specifically, if an industry is supported by the government with export subsidies or distorting administrative measures alike, the TC index and RCA index of the industry may rose distinctly, but the actual profitability of the industry remains essentially unchanged [39]. In contrast, the *VRCA* index can overcome this shortcoming of the traditional comparative advantage indices.

### 3.3. Indicators of Econometric Regression 

#### 3.3.1. Environmental Regulations (ER)

Environmental regulation policies are equivalent to the implementation of additional constraints on production behaviors of enterprises [40]. An industry with more stringent environmental regulation policies expends more on reduction in pollution emissions. Hence, such expenditure, to a large extent, reflects the efficacy of an industry’s environmental regulation policies and should be used to measure the stringency of such regulations [41]. Compared with other indices, including the pollution levy and emissions standards charged or formulated by the government, industrial expenditure on pollution discharge control can better reflect the actual intensity of emissions reduction [42]. Therefore, this study measures the stringency of environmental regulations by an industry’s expenditure on the treatment of industrial wastewater and waste gas as a percentage of its value added. Furthermore, a robustness check is conducted by calculating an industry’s expenditure on the treatment of industrial wastewater and waste gas as a percentage of the industry’s main operational costs.

#### 3.3.2. Other Control Variables 

Technological innovation is the key driver of the total factor productivity and competitiveness of an industry. Technological innovation within enterprises is usually undertaken through either independent R and D or technology introduction [7]. In this study, the former (*RD*) is measured by an industry’s internal expenditure on scientific and R and D activities as a percentage of gross industrial output, while the latter (*TI*) is measured by expenditure on technology introduction as a percentage of gross industrial output.

Capital intensity (*Capital*) is a crucial determinant influencing export competitiveness. A higher level of capital intensity indicates that the industry possesses more advanced equipment and tends to enjoy a competitive advantage. In this study, capital intensity is evaluated by the ratio of industrial fixed-asset investments to the number of employees in industrial units (10,000 RMB/person) [25].

Human capital (*Human*) is another key determinant of export competitiveness. Increasing investment in human capital can enhance labor skills and the absorptive capacity for international technology spillovers, thus improving the export competitiveness of an industry [43]. At present, the indices used for measuring human capital are diverse and inconsistent, including indices such as wage level, years of schooling, and the percentage of scientific and technical personnel. Due to price distortions in China’s factor market, wage levels do not realistically reflect the conditions of an industry’s human capital. Hence, in this study, human capital is measured by the ratio of scientific and technical personnel to employees in an industry.

An increase in the size of an enterprise (*Size*) can lead to economies of scale and economies of scope, favoring innovation and competitiveness [21]. In this study, the size of the enterprise is measured by the ratio of gross industrial output to the number of enterprises in the respective industry.

Foreign direct investment (*FDI*) can facilitate the technological advancement of enterprises through international technology spillovers, thereby improving the production processes and quality of products and lifting their international competitiveness [44]. This study measures the level of foreign direct investment of an industry by the ratio of the total gross industrial output across foreign-funded enterprises and enterprises funded by Hong Kong, Macao, and Taiwan.

### 3.4. Sources of Data

Since 2000, the Chinese government has introduced a series of laws and regulations for environmental protection, including the Detailed Rules for the Implementation of the Law of the People’s Republic of China on the Prevention and Control of Water Pollution, the Law of the People’s Republic of China on the Prevention and Control of Atmospheric Pollution, and the Law of the People’s Republic of China on the Prevention and Control of Environmental Pollution by Solid Waste. This marks a transition toward increasingly stringent environmental regulation. The World Input-Output Database (WIOD), necessary for measuring *VRCA*, was last updated in 2014; therefore, the sample period for this study is 2000 to 2014.

The input-output data used in the estimation of export value added was obtained from Eurostat’s WIOD. The latest version, released in 2016, provided the non-competitive world input-output tables, supply and use tables, and socio-economic accounts covering 56 industries across 43 countries from 2000 to 2014, facilitating the measurement and analysis of value added and international comparisons in this study. *RIM* data was sourced from the Trade in Value Added (TiVA) Database of OECD Stat, in which the EXGR_RIM table offered annual data on the domestic value added content of reimported intermediate goods in exports by industry across the sample countries and regions surveyed. This study drew inspiration from the industrial classification by WIOD and OECD Stat and derived China’s *RIM* by industry accordingly. Data on the world’s export value added by industry and total export value added were extracted from the domestic value added content in gross exports (EXGR_DVA) table of OECD Stat.

To gauge the stringency of environmental regulation, industrial data regarding expenditure on the treatment of industrial wastewater and waste gas were obtained from China Environmental Statistical Yearbook, while data on industrial value added and the costs of main operations were from China Industry Statistical Yearbook. As the statistical specifications adopted in China Environmental Statistical Yearbook were revised in 2001, three key assumptions were made: (1) In 2000, the stringency of environmental regulation was consistent across three sub-industries; namely, food processing and manufacturing, beverage manufacturing, and tobacco manufacturing; (2) The stringency remained the same in 2000 and 2001 across four sub-industries: the manufacturing of garments and other fiber products; timber processing and the manufacturing of bamboo, cane, palm fiber, and straw products; furniture manufacturing; and the manufacturing of cultural, educational, and sporting goods; and (3) In 2000, the stringency was consistent across six sub-industries: the manufacturing of ordinary machinery; special purpose equipment; transport equipment; electric equipment and machinery; electronic and telecommunications equipment; and instruments, meters, cultural, and office equipment. In addition, due to missing data on the industrial value added in 2004, interpolation was carried out by multiplying the gross industrial output in 2004 by the average percentage of value added in gross industrial output across the two adjacent years. The industrial value added between 2008 and 2014 was estimated based on the relevant growth rate published by the National Bureau of Statistics of China. Data regarding the other control variables were retrieved from China Statistical Yearbook on Science and Technology and China Industry Statistical Yearbook.

The above-mentioned databases have adopted differing standards for industrial classification and statistical specifications. Data from WIOD and OECD Stat are primarily based on the ISIC classification standard, while Chinese industries are classified according to the Industrial Classification for National Economic Activities. It was, therefore, necessary to integrate the two types of data. With reference to the practice used in previous research, this study coupled the Industrial Classification for National Economic Activities (2002) with ISIC (Rev. 4) and partitioned the manufacturing industry into 27 sub-industries (excluding the recycling and disposal of waste due to a substantial gap in data). Additionally, this study selected the year 2000 as the sample baseline and utilized winsorization to trim the top and bottom 1% of the sample data.

The descriptive statistics of the main variables are enumerated in Table 1. Then we measure the effects of environmental regulation on export competitiveness and examine the different mechanisms through which environmental regulation acts on export competitiveness based on Equation (1) and Equation (2), respectively.

## 4. Results

### 4.1. Benchmark Regression Results

To eliminate the effects of multicollinearity, a pooled ordinary least squares (POLS) estimation is applied to Equation (1) through stepwise regression on panel data. The resulting estimates are reported in Table 2. While no control variables are added to column (1), other control variables and the industry fixed effect are gradually added in columns (2) to (6). The estimated coefficients of the core explanatory variable ln*ER* are significantly positive at the 5% significance level, with or without the control variables. This suggests that boosting the stringency of environmental regulation could significantly improve the export competitiveness of China’s manufacturing industry. Specifically, when other control variables are excluded, every 1% increase in the stringency of environmental regulation is associated with a 2.9% increase in the export competitiveness of the manufacturing industry (*VRCA*). When all the control variables are included, environmental regulation has a promotion effect of approximately 2% on export competitiveness. This conclusion supports the Porter hypothesis. The coefficients of the quadratic term of environmental regulation are significantly positive, which shows that environmental regulation has a non-linear effect on the export competitiveness of the manufacturing industry; a gradual increase in the stringency of environmental regulation first inhibits and subsequently promotes export competitiveness, thus forming a dynamic “U-shaped” effect. That is, the effect of environmental regulation on the export competitiveness of the manufacturing industry exhibits a turning point. Before this turning point is reached, environmental regulation has a significant cost effect that inhibits competitiveness; however, once this point is exceeded the offset effect begins to dominate, facilitating an improvement in export competitiveness. This indicates that certain prerequisites must be fulfilled to validate the Porter hypothesis.

In terms of the other control variables, both the estimates of independent R and D (ln*RD*) and technology introduction (ln*TI*) are significantly positive, indicating that both types of technological innovation significantly promote the export competitiveness of the manufacturing industry. In comparison, however, the promotion effect of technology introduction surpasses that of independent R and D; the estimated coefficients, as shown in column (6), are 0.055 and 0.009, respectively. This is expected because technology introduction is a direct means to facilitate technological advancement and, consequently, improve the export competitiveness of the manufacturing industry. Independent R and D, however, usually requires a longer R and D cycle to play its role effectively. The estimated coefficients of capital intensity (ln*Capital*) are significantly negative, which shows that, in contrast to expectations, a higher capital-labor ratio diminishes the export competitiveness of the manufacturing industry. This finding could be interpreted by the appropriate technology theory proposed by Lin and Zhang [45]. At present, China is still considered a labor-endowed country based on its structure of factor endowment. A higher level of human capital intensity propels China farther from the most appropriate technology structure and constitutes a hindrance to the improvement in comparative trade advantage. The estimated coefficients of human capital (ln*Human*) are significantly positive in line with expectations and demonstrates that increasing the number of science and technology personnel as a proportion of total employees may contribute to the comparative advantage of the industry. The estimated coefficients for size of the enterprise (ln*Size*) are positive, albeit insignificantly. This shows that the size of an enterprise is not a key factor influencing the export competitiveness of China’s manufacturing industry. Moreover, since the reform of the Chinese economic system, small private enterprises have occupied an ever-growing portion of the market and this has not resulted in a decline in export competitiveness of China’s manufacturing industry. The estimated coefficients for foreign direct investment (ln*FDI*) are significantly positive, which indicates that the introduction of foreign investment is a key contributor to the export competitiveness of China’s manufacturing industry.

Moreover, the relationships among environmental regulation, technological innovation, and export performance may show dynamical changes in different phases according to the available literature. There is a “U-shaped” relationship between environmental regulation and export performance, and environmental regulation has an adverse effect on export performance of China’s industrial enterprises over the period 2005–2009 [46]. This finding reflects that the stringency of environmental regulation in China is to the left of the inflection point and environmental policies are weakly enforced in that period. And then, it is demonstrated that China’s environmental policies are significantly conducive to technological innovation in industry from 2006 to 2015 [47]. Industrial innovation appears to be a shift to green technology innovation [48], and the technology innovation efficiency in China’s manufacturing industry shows an upward trend during 2003-2016 [49]. In addition, the innovation efficiency in high-end manufacturing industry has come up to the middle and high levels, and the competitiveness has increased substantially over the period 2012–2017 [50]. Combined with the above results of this paper, it is observed that environmental regulation in China is enforced much more strictly at present, consequently stimulating technological innovation and promoting the export competitiveness of manufacturing industry.

### 4.2. Heterogeneity Analysis

As manufacturing sub-industries vary in industrial attributes and characteristics, particularly in the intensity of pollution emissions, environmental regulation may display distinctive heterogeneity in its effect on the export competitiveness of different industries. Thus, this study divides the sample sub-industries into groups for regression and examines the heterogeneous effects of environmental regulation on export competitiveness. Existing research derives the intensity of pollution emissions by industry through linear standardization and divides manufacturing sub-industries accordingly into lightly-, moderately-, and heavily-polluting sub-industries [41]. With reference to the industrial classification method proposed by Cheng et al. [41], this study re-estimates the export competitiveness of sub-industries using these three levels of pollution intensity. The estimated outcomes for the subsamples are listed in Table 3, where columns (1) and (2), (3) and (4), and (5) and (6) represent the estimates for lightly, moderately, and heavily polluting sub-industries, respectively. Each of these sample categories considers two sets of scenarios: the inclusion and exclusion of control variables and the industry fixed effect.

The estimates for the three types of sub-industries reveal that the environmental regulation coefficients for lightly polluting sub-industries are positive, albeit insignificantly, while the coefficients for moderately and heavily polluting sub-industries are significant and positive, with the former (ln*ER* = 0.041 in column (4)) lower than the latter (ln*ER* = 0.083 in column (6)). This implies that the promotion effect of environmental regulation on the export competitiveness of China’s manufacturing industry is mainly evident in moderately and heavily polluting sub-industries—more significantly in the latter than the former—and insignificant in lightly polluting sub-industries. Likewise, as shown by the quadratic-term coefficients, the “U-shaped” effect of environmental regulation on export competitiveness is also concentrated in moderately and heavily polluting sub-industries.

In fact, there exists evident heterogeneity in pollution intensity and economic development characteristic of China’s manufacturing industry [41]. For instance, the production process of heavily polluting sub-industries is generally accompanied by high energy consumption and industrial wastewater or waste gas discharge. At present, the environmental protection system with stringent standards has been implemented in China, in order to effectually achieve the targets of energy conservation and emission reduction. Consequently, compared with lightly polluting sub-industries, heavily polluting sub-industries are confronted with much larger survival pressure. Therefore, enterprises in heavily polluting sub-industries have to promote the recycling of resources and strengthen technological innovation, so as to improve their production efficiency and market competitiveness. According to the results above, we can infer that China’s environmental policies play an important role in sustainable development of ecological environment and foreign trade.

### 4.3. Robustness Check

The above sections examine the effect of environmental regulation on the export competitiveness of China’s manufacturing industry and its heterogeneity according to different types of industries. However, regression Equation (1) may be subject to endogeneity due to omitted variables. In addition, while environmental regulation is measured by an industry’s expenditure on the treatment of industrial wastewater and waste gas as a percentage of its value added, the data on industrial value added is incomplete in some years and inferred based on the growth rate. This could result in estimation bias. Therefore, to ensure the reliability of the estimated results, a robustness check is conducted from two perspectives.

First, to alleviate the effects of endogeneity, the panel data of the manufacturing industry is subject to SYS-GMM estimation. AR (1), AR (2) autoregressive models, and Sargan’s test are adopted to determine the validity of instrumental variables and test for over-identification. The estimated results in Table 4 show that, after controlling for endogeneity, the promotion effect of environmental regulation on the export competitiveness of China’s manufacturing industry remains significant (column (1) in Panel A). No substantive change is observed in the estimated coefficients of the three industrial categories and their significance (columns (2)–(4) in Panel A). Furthermore, the results of AR (1) and AR (2) tests show that, at the 10% significance level, the finite differences of random error terms across all the estimation models follow first-order but not second-order autocorrelation. The results of Sargan’s test suggest that, at the 10% significance level, none of the estimation models reject the null hypothesis that the over-identifying restrictions are valid. Hence, it is concluded that the SYS-GMM estimation in this study has yielded consistent and reliable results.

Second, Equation (1) is re-calculated with the stringency of environmental regulation measured by expenditure on the treatment of industrial wastewater and waste gas as a percentage of an industry’s main operational costs. The resulting estimates are reported in Panel B of Table 4. It is found that, after altering the measure of environmental regulation, the estimates of environmental regulation and the quadratic terms, both in the full sample and the subsamples, remain consistent with the above benchmarking regression results.

This section attests to the robustness and reliability of the results in benchmark regression and heterogeneity analysis. Further, we conduct mechanism analysis to investigate the internal relation among environmental regulation, technological innovation, and export competitiveness.

### 4.4. Mechanism Analysis

Most existing studies agree that environmental regulation affects export competitiveness through the mechanisms of cost effect and offset effect on technological innovation [6]. Nonetheless, there are several differing conclusions in the literature. A possible reason is that enterprises are free to choose between independent R and D and technology introduction [7], which are influenced by environmental regulation differently. To test this, regression analysis is applied to Equation (2) to test the mechanisms through which environmental regulation acts on the export competitiveness of the manufacturing industry. The regression results are illustrated in Table 5.

The following section focuses on the estimated coefficients of the interaction terms between environmental regulation and the two types of technological innovation. According to the results of POLS estimation shown in Column (1), the estimated coefficient of the interaction term lnER×lnRD is significant and negative, while that of lnER×lnTI is significant and positive. This indicates a clear difference in the effect of environmental regulation depending on the mechanism of technology innovation; environmental regulation influences independent R and D in the manufacturing industry primarily through the cost effect, which inhibits export competitiveness, while it influences technology introduction through the innovation offset effect, which favors export competitiveness.

This finding may be attributed to China’s approach to technological innovation, which is to ease the introduction of existing technologies coupled with the difficulty to innovate. By embarking on technological transformation through technology introduction, enterprises can obtain faster returns on innovation performance, while increased inputs to R and D innovation do not necessarily translate to satisfactory innovation performance. It is also necessary to consider the combined effects of factors including the stage of industrial development, industrial characteristics, and resource endowment conditions of the industry [51]. Therefore, when compelled by the government’s environmental regulation policies to undertake technological innovations, enterprises are inclined toward technology introduction. Given that the total amount of an enterprise’s resources is fixed, independent R and D will be subject to the crowding-out effects of both the costs of environmental regulation and expenditure on technology introduction. This explains the opposing effect of environmental regulation on the export competitiveness of the manufacturing industry via independent R and D and technology introduction.

In addition, column (2) displays the results of SYS-GMM estimation, and column (3) illustrates the results of POLS estimation in which the stringency of environmental regulation is measured by expenditure on the treatment of industrial wastewater and waste gas as a percentage of an industry’s main operational costs. The results show that, after altering the regression technique and adjusting the measure of the core explanatory variable, the estimated outcomes of the two interaction terms remain robust.

## 5. Discussion

A current and universal challenge, particularly in developing nations, is the establishment of effective environmental regulation policies that protect the ecological environment without adversely affecting the international competitiveness of the domestic manufacturing industry. To deal with this dilemma, this study examines the effect of environmental regulation on the export competitiveness of China’s manufacturing industry from the standpoint of export value added. The findings reveal that environmental regulation has a “U-shaped” relationship with export competitiveness, and significantly improves the RCA based on export value added of China’s manufacturing industry. Thus, it’s suggested that China’s environmental policies are increasingly strengthened, and the stringency of environmental regulation has come up to the right of the inflection point over the period 2000–2014, with the innovation offset effect greater than the compliance cost effect. Therefore, we confirm that well-designed environmental regulation can achieve a win-win situation of ecological environment and foreign trade, which is basically in accord with the research of Song and Sung [52] based on South Korea’s manufacturing industry. For China, implementation of environmental regulation increasingly favors the coordinated development of environment and foreign trade. Stringent policies will compel enterprises to engage in technological innovation and refine their production processes, allowing them to improve their competitiveness through environmentally-sustainable production processes.

However, further results of heterogeneity analysis imply that the positive effect of environmental regulation on export competitiveness is only reflected in heavily and moderately polluting sub-industries, but an insignificant effect in lightly polluting sub-industries. A possible explanation is that lightly polluting sub-industries are subject to less stringent environmental regulation and fail to achieve a strong innovation offset effect [46]. Similarly, Cheng et al. [41] test the Porter’s hypothesis based on a comparative analysis of sub-industries under different pollution intensities, and find that heavily polluting industry is at the forefront of technological innovation in China’s manufacturing industry. Marconi [53] indicates that the RCA of 14 EU countries in both water-polluting industries and air-polluting industries remains stable or is enhanced significantly, while the competitiveness of the more clean industries is in decline. Additionally, it is found that the impacts of environmental performance on employment are also distinctly different across clean industries and dirty industries in China [54,55]. All these findings confirm that the effects of environmental regulation may be obviously heterogeneous in different sub-industries, which suggests that the government should consider the heterogeneous characteristics of sub-industries and propose appropriate environmental regulatory design. Specifically, when formulating environmental regulation policies, the government should adopt levels of stringency suited to the various levels of pollution emissions intensity, to encourage an innovation offset effect throughout the industry.

Afterwards, based on the results of mechanism analysis, it can be indicated that two approaches to technological innovation have significantly different responses to environmental regulation. To be specific, stringent environmental regulation restrains the independent R and D of China’s manufacturing industry, thus limiting export competitiveness; in contrast, it stimulates technology introduction, and then improves the export competitiveness of the manufacturing industry. However, it should be pointed out that both technological innovation and transformation ability are the critical factors that determine the competitiveness of manufacturing industry [56]. China, as a developing country, can directly introduce the new inventions and technology from developed countries, and then accomplish the application of technology transformation and industrialization with the improvement of international competitiveness. While independent R and D in China, such as basic research and technology that have increased dramatically, may run into a serious problem of “chain breakage” that is from scientific findings to engineering application, and then weaken the competitiveness of the manufacturing industry [56]. Nonetheless, there are also some different views on this issue. The long-term direct introduction of advanced technologies is likely to lead to serious technology dependence, thereby possibly restricting the improvement of industrial innovation capacity [57]. In contrast, independent R and D may provide a steady stream of motivation and sustainably promote the economic growth [58]. Therefore, the government should support the independent R and D activities of enterprises more vigorously to counterbalance the cost effect of environmental regulation. This may encourage independent innovation in core technologies and process designs, and then overcome the dependence on technology introduction, thus improving comprehensive technology innovation and outcome transformation abilities, and providing new competitive advantages for Chinese export products.

Moreover, there are other factors that play key roles in improving the export competitiveness of China’s manufacturing industry and the coordinated development of environment and foreign trade. Firstly, on the basis of the results above, the introduction of foreign direct investment contributes to the improvement of the RCA based on export value added. Similarly, Feng et al. [59] confirm that the two-way foreign direct investment is significantly beneficial to the green innovation efficiency of China’s manufacturing industry. Therefore, we believe that the extroversion and internationalization of China’s manufacturing industry, together with bilateral investment agreements in an international context, can favor the export competitiveness of industrial enterprises through environmentally friendly production processes. Secondly, due to the key role of involvement of local small and medium-size enterprises (SMEs), the size of an enterprise is not a critical factor influencing the export competitiveness. SMEs are more competent to integrate the target of the functional departments into the innovation activities [60], and the market and competitive mechanisms for SMEs may encourage innovators to continuously breakthrough innovation [30]. Additionally, e-commerce and management of information systems (MIS), as important supports of the new era of internet economy, are also conducive to promoting the competitiveness of SMEs. Thirdly, a higher capital intensity leads to a weaker export competitiveness, because enterprises with high capital intensity have to increase their spending on pollution abatement [61]. Generally, an additional use of capital in production process may result in increased energy consumption and pollution emission [52]. Lastly, as an important endogenous driving force of economic growth, human capital plays a crucial part in accumulating knowledge and skills, thus improving the productivity and competitiveness of manufacturing industry. This is also backed up by the results of this study.

## 6. Conclusions

Based on the data of 27 sub-industries from 2000 to 2014, this study investigates the export competitiveness of China’s manufacturing industry from the viewpoint of export value added. Furthermore, the Porter hypothesis is revisited for an empirical investigation of the effects of environmental regulation on export competitiveness and to determine the presence of intra-industry heterogeneity. Afterwards, two approaches to technological innovation are also adopted to unravel the mechanisms through which environmental regulation influences export competitiveness. The main conclusions we have drawn are as follows: (1) Environmental regulation has a promotion effect of approximately 2% on the export competitiveness of China’s manufacturing industry; however, this effect is non-linear and displays a “U-shaped” tendency, indicating that certain prerequisites must be fulfilled to validate the Porter hypothesis. (2) In addition, significant intra-industry heterogeneity exists in the degree to which environmental regulation affects export competitiveness, with a substantial effect evident primarily in heavily polluting sub-industries and secondarily in moderately polluting sub-industries but an insignificant effect in lightly polluting sub-industries. (3) Environmental regulation also differs significantly in the mechanisms through which it affects different approaches to technological innovation; independent R and D is affected by environmental regulation through the cost effect, which limits export competitiveness, while technology introduction is affected through the innovation offset effect, which favors export competitiveness. These conclusions retain their robustness after controlling for endogeneity and altering the measurement of the core explanatory variable.

However, it should be noted that this paper has some limitations, hereby providing directions for future research. Although the industrial heterogeneity and different approaches to technological innovation have been taken into account in this study, there are different types of environmental regulations as well, which may have heterogeneous effects on technological innovation and export competitiveness. In addition, enterprises of different ownership differ in innovation characteristics due to different incentive mechanisms [41], but it is difficult to identify the ownership with the industry data used in this paper. Additionally, these are definitely worth further research in future.

## Figures and Tables

**Table 1:** Statistical descriptions of the main variables.

Variable Category	Variable	Description	Observations	Mean	SD
Dependent variable	ln*VRCA*	RCA index based on export value added	405	0.363	0.589
Independent variable	ln*ER*	Environmental regulations	405	−5.146	1.471
Control variables	ln*RD*	Independent R and D	405	−5.003	0.815
	ln*TI*	Technology introduction	405	−6.726	1.676
	ln*Capital*	Capital intensity	405	2.288	0.780
	ln*Human*	Human capital	405	−3.309	0.911
	ln*Size*	Size of enterprise	405	−0.104	0.983
	ln*FDI*	Foreign direct investment	405	−1.327	1.098

Note: The statistical descriptions in Table 1 are based on the logarithms of the variables.

**Table 2:** Benchmark regression results.

Variable	(1)	(2)	(3)	(4)	(5)	(6)
ln*ER*	0.029 ***	0.025 **	0.027 **	0.022 **	0.028 ***	0.020 ***
	(3.064)	(2.047)	(2.217)	(2.303)	(3.611)	(4.038)
(ln*ER*) ^2^		0.005 **	0.006 *	0.005 **	0.003 ***	0.004 **
		(2.102)	(1.742)	(2.014)	(3.113)	(2.010)
ln*RD*			0.011 **	0.013 ***	0.011 **	0.009 **
			(2.353)	(3.428)	(2.021)	(2.050)
ln*TI*			0.054 **	0.052 ***	0.047 ***	0.055 **
			(2.144)	(3.773)	(2.904)	(3.660)
ln*Capital*				−0.076 *	−0.058 *	−0.063 *
				(−1.714)	(−1.722)	(−1.800)
ln*Human*					0.037 **	0.028 *
					(2.077)	(1.793)
ln*Size*					0.084	0.097
					(0.464)	(1.307)
ln*FDI*						0.103 **
						(2.029)
*Constant*	1.463 ***	−3.428 ***	−1.753 **	0.668 ***	3.742 **	−2.563 ***
	(3.172)	(−4.035)	(−3.429)	(2.885)	(2.111)	(−3.380)
Industry fixed effect	N	Y	Y	Y	Y	Y
Observations	405	405	405	405	405	405
R-squared	0.1009	0.1050	0.1117	0.1145	0.1163	0.1220

Notes: Robust t-statistics are in parentheses; *, **, and *** represent 10%, 5%, and 1% significant levels, respectively; N indicates that the industry fixed effect is not controlled, whereas Y denotes that the industry fixed effect is controlled.

**Table 3:** Estimated outcomes according to subsample.

Variable	(1)	(2)	(3)	(4)	(5)	(6)
ln*ER*	0.007	0.005	0.048 **	0.041 **	0.089 **	0.083 ***
	(0.547)	(1.002)	(2.041)	(2.215)	(2.030)	(3.142)
(ln*ER*)^2^		0.002		0.008 ***		0.007 **
		(0.336)		(3.422)		(2.183)
*Control Variables*	N	Y	N	Y	N	Y
Industry fixed effect	N	Y	N	Y	N	Y
Observations	150	150	120	120	135	135
R-squared	0.037	0.096	0.122	0.153	0.081	0.187
Sample category	Lightly polluting sub-industries		Moderately polluting sub-industries		Heavily polluting sub-industries	

Notes: Robust t-statistics are in parentheses; *, **, and *** represent 10%, 5%, and 1% significant levels, respectively; N indicates that the control variables are excluded or that the industry fixed effect is not controlled; Y denotes that all the control variables are included or that the industry fixed effect is controlled. The constant term is included in the regression in all the columns.

**Table 4:** Results of the robustness check

Variable	(1)	(2)	(3)	(4)
Panel A: SYS-GMM estimation				
ln*ER*	0.014 ***	0.004	0.057 ***	0.073 ***
	(3.137)	(0.953)	(3.372)	(2.938)
(ln*ER*)^2^	0.009 *	0.011	0.020 *	0.006 **
	(1.849)	(1.342)	(1.743)	(2.005)
AR (1)	[0.034]	[0.057]	[0.073]	[0.020]
AR (2)	[0.214]	[0.363]	[0.367]	[0.188]
Sargan test	[0.838]	[0.874]	[0.728]	[0.752]
Panel B: Altering the measure of environmental regulation				
ln*ER*	0.163 **	0.026	0.209 **	0.306 ***
	(2.235)	(1.248)	(2.046)	(3.281)
(ln*ER*)^2^	0.016 **	0.003	0.015 *	0.024 **
	(2.163)	(0.794)	(1.832)	(2.227)
R-squared	0.238	0.176	0.147	0.124
*Control Variables*	Y	Y	Y	Y
Industry fixed effect	Y	Y	Y	Y
Observations	405	150	120	135
Sample category	Full sample	Lightly polluting sub-industries	Moderately polluting sub-industries	Heavily polluting sub-industries

Notes: Robust t-statistics are in parentheses; *, **, and *** represent 10%, 5%, and 1% significant levels, respectively; N indicates that the control variables are excluded or that the industry fixed effect is not controlled; Y denotes that all the control variables are included or that the industry fixed effect is controlled; the reported results of AR (1), AR (2), and Sargan’s test are the *p* values of the respective statistics; the constant term is included in the regression in all columns.

**Table 5:** Results of tests on influencing mechanisms.

Variable	(1)	(2)	(3)
ln*ER*	0.018 **	0.012 **	0.155 **
	(2.026)	(2.107)	(2.272)
(ln*ER*)^2^	0.004 *	0.007 *	0.010 **
	(1.913)	(1.844)	(2.306)
ln*ER* × ln*RD*	−0.0043 **	−0.0029 *	−0.0062 **
	(−2.129)	(−1.860)	(−2.145)
ln*ER* × ln*TI*	0.0011 ***	0.0005 **	0.0014 **
	(3.058)	(2.145)	(2.331)
*Control Variables*	Y	Y	Y
Industry fixed effect	Y	Y	Y
AR (1)	-	[0.017]	-
AR (2)	-	[0.483]	-
Sargan’s test	-	[0.934]	-
Observations	405	405	405
R-squared	0.215	-	0.288

Notes: Columns (1) and (2) show the results of POLS estimation and SYS-GMM estimation respectively, while column (3) displays the results of POLS estimation in which the stringency of environmental regulation is measured by expenditure on the treatment of industrial wastewater and waste gas as a percentage of the industry’s costs of main operations. Robust t-statistics are in parentheses; *, **, and *** represent 10%, 5%, and 1% significant levels, respectively; N indicates that the control variables are excluded or that the industry fixed effect is not controlled; Y denotes that all the control variables are included or that the industry fixed effect is controlled; the reported results of AR (1), AR (2), Sargan’s test results are the *p* values of the respective statistics; the constant term is included in the regression in all columns.
